# Comparison of femoral neck system and three cannulated cancellous screws in the treatment of vertical femoral neck fractures: clinical observation and finite element analysis

**DOI:** 10.1186/s12938-023-01083-1

**Published:** 2023-03-02

**Authors:** Shaolong Huang, Yazhong Zhang, Xu Zhang, Chengqiang Zhou, Wenbo Li, Yunqing Wang, Bin Wang, Ziqiang Zhu

**Affiliations:** 1 Department of Orthopedics, The Second Affiliated Hospital of Xuzhou Medical University, No 32 Meijian Road, Xuzhou, 221000 Jiangsu China; 2 Graduate School of Xuzhou Medical University, Xuzhou, 221000 Jiangsu China

**Keywords:** Femoral neck system, Cannulated cancellous screws, Vertical femoral neck fracture, Finite element analysis, Surgical fixation devices

## Abstract

**Objective:**

The purpose of this study was to compare the biomechanical and clinical results of two surgical methods for the treatment of vertical femoral neck fractures: Femoral neck system (FNS) and traditional three cannulated cancellous screws (CCS).

**Methods:**

First, we developed three different vertical femoral neck fracture models for the finite element analysis, with angles of 55°, 65°, and 75°, respectively. Two experimental groups were set up: the FNS group and the CCS group. Each fracture group was tested under axial loads of 2100 N to measure the femur's displacement, Von Mises stress (VMS), and its internal fixation components. Secondly, we retrospectively included the cases of vertical femoral neck fractures with FNS and CCS in our hospital from May 2019 to May 2021. In this study, we compared the duration of intraoperative fluoroscopy, operative time, hospital stay, fracture healing time, Hemoglobin loss, Harris score of hip joint function, and postoperative complications among patients undergoing hip joint replacement.

**Results:**

In terms of finite element analysis, FNS has better anti-displacement stability than CCS at 55°and 65°, while FNS is greater than CCS in Von Mises stress. Clinically, we followed up on 87 patients for an average of 12 months. FNS was superior to traditional CCS in fracture healing time, operation time, fluoroscopy duration, fracture healing time, and Harris hip function score.

**Conclusion:**

FNS is superior to traditional CCS in biomechanical and clinical aspects of treating vertical femoral neck fractures. There is potential for FNS to become a new treatment option for vertical femoral neck fractures.

## Introduction

Femoral neck fractures are currently the most common clinical fracture type, accounting for 3.6% of systemic fractures and 57% of hip fractures. The incidence of femoral neck fractures is increasing, and their onset is no longer limited to the elderly, especially vertical fractures caused by high energy [1]. One of the most popular clinical classification systems for femoral neck fractures is Pauwels classification, which was initially introduced in 1935 [2, 3]. Pauwels III means that the Pauwels angle is greater than 50° [4, 5]. Vertical fractures of the femoral neck (Pauwels III) in young people are challenging to treat and are usually the result of high-energy trauma. High-energy injury can easily cause severe bone comminution of the femoral neck, which makes fracture reduction and fixation more difficult. These injuries increase the risk of complications, such as fixation failure, malunion, nonunion, and bone erosion [6, 7].

Current internal fixation methods include hollow compression screws, dynamic hip screws, plates, and the new compression locking nail board system. Even though fixed implants have made significant strides, the proper management of these injuries is still debatable due to issues with internal fixation device loosening, loosening of nail backs, and weak anti-rotation force in all types of procedures [8–12]. Since postoperative complications are often severe, doctors are examining minimally invasive implantation to reduce injuries and complications during surgery. This type of injury does not currently have a consensus regarding the best type of implant.

Three cannulated cancellous screws have become the most common fixation device because of their advantages, such as linear dynamic compression during weight-bearing, less invasive surgery, less bleeding, and shorter hospital stay [13]. However, for more axial and rotationally unstable vertical femoral neck fractures, when fixed with ordinary cannulated cancellous screws (CCS), strong shear force in the hip often leads to fixation failure. The total incidence of complications ranges from 20 to 86% [7]. Femoral Neck System (FNS), a new type of femoral neck fracture treatment device, has the advantages of anti-rotation, angle stability, dynamic fixation, and minimally invasive surgery [14, 15]. Our previous study demonstrated the effectiveness of FNS during short follow-up [16], suggesting it could be the preferred device for treating vertical femoral neck fractures in the future.

This study achieved the following objectives by constructing a vertical femoral neck fracture model and collecting clinical data of patients with vertical femoral neck fracture: 1. Finite element analysis method was used to analyze and compare the stability of shear and rotational forces of FNS and CCS in the treatment of vertical femoral neck fracture. 2. The clinical efficacy of FNS and CCS was evaluated and compared in treating vertical femoral neck fractures in young adults. 3. The long-term stability and complication rate of FNS and CCS were compared.

### Statistical analysis

SPSS 23.0 version (SPSS Inc. Chicago, IL, USA) was used for statistical analysis. The failure of fixation (screw loosening, screw prolapse), the degree of shortening of the femoral neck, nonunion, and osteonecrosis of the femoral head (expressed as a percentage (%)) were analyzed by the Chi-square test. Operation time, hemoglobin loss, fluoroscopy time, hospitalization time, fracture recovery time, hospitalization cost, and Harris score are all expressed as mean ± standard deviation and were analyzed by independent sample t-test.

## Results

### Finite element analysis results of two surgical methods

#### Displacement of the femur

**Fig. 1:**
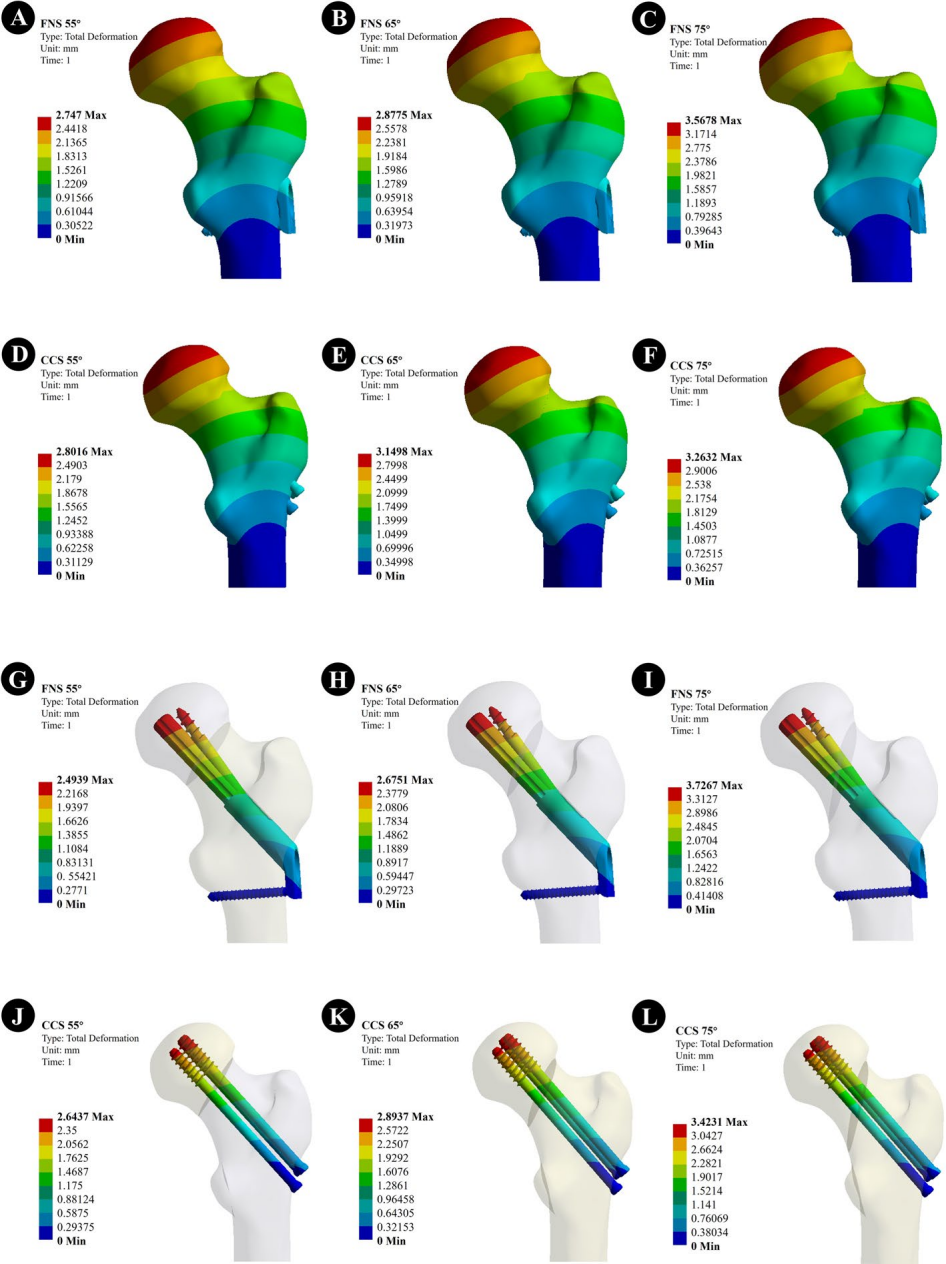
The displacement of the femur and the internal fixation; **A**–**C** The displacement of the femur of the FNS model with Pauwels angles of 55°, 65°, and 75°; **D**–**F** The displacement of the femur of the CCS model with a Pauwels angles of 55°, 65°, and 75°; **G**–**I** The displacement of the internal fixation of FNS model with Pauwels angles of 55°, 65°, and 75°; **J**–**L** The displacement of the internal fixation of CCS model with Pauwels angles of 55°, 65°, and 75°

According to the displacement profile of the Pauwels fracture at 55°, 65°, and 75°, the maximum displacement occurred in the upper part of the femoral head. When the Pauwels angle was 55°, the maximum displacement of the femur of FNS was 2.747 mm, while that of the CCS group was 2.801 mm. When Pauwels angle was 65°, the maximum displacement of the femur of FNS was 2.877 mm, and that of the CCS group was 3.149 mm. When Pauwels angle was 75°, the maximum displacement of the femur of FNS was 3.567 mm, and that of the CCS group was 3.263 mm (Fig. 1).

#### Displacement of the internal fixation implant

The maximum displacement of the internal fixation occurred at the top of the screw. When the Pauwels angle was 55°, the maximum displacement of the internal fixation component of FNS was 2.493 mm, and that of CCS was 2.643 mm. When the Pauwels angle was 65°, the maximum displacement of the internal fixation components was 2.675 mm and that of the CCS group was 2.893 mm. When Pauwels angle was 75°, the maximum displacement of FNS internal fixation components was 3.726 mm and that of the CCS group was 3.423 mm (Fig. 1).

#### Von Mises stress (VMS) of internal fixation components

**Fig. 2:**
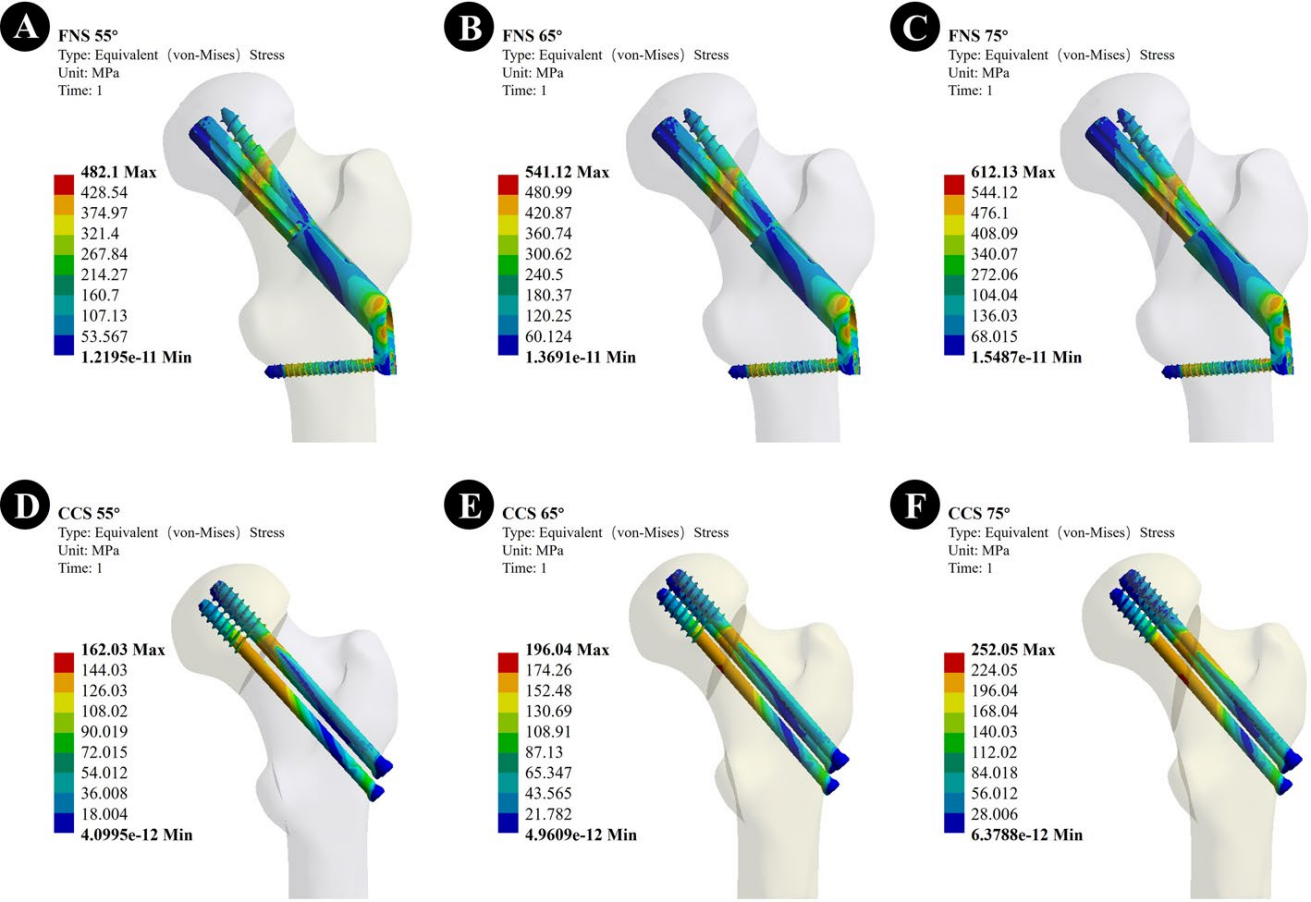
The VMS of the internal fixation; **A**–**C** The VMS of the internal fixation of the FNS model with Pauwels angles of 55°,65°, and 75°; **D**–**F** The VMS of the internal fixation of the CCS model with Pauwels angles of 55°,65°, and 75°

The VMS peak values of the internal fixation assembly were as follows: when the Pauwels angle was 55°, the VMS peak values of the internal fixation assembly of FNS were 482.1 MPa and CCS was 162.03 MPa. When the Pauwels angle was 65°, the VMS peak of the FNS internal fixation module was 541.12 MPa and the CCS was 196.04 MPa. When the Pauwels angle was 75°, the VMS peak of the FNS internal fixation module was 612.13 MPa and the CCS was 252.05 MPa. In the CCS assembly, the VMS was concentrated on the surface of the screw near the fracture line and distributed evenly along the screw. In the FNS group, the VMS was more concentrated at the junction of the sliding hip screw and the anti-rotation screw and distributed evenly along the screw (Fig. 2).

### Clinical results of two kinds of surgery

#### Patient characteristics

There was no significant difference between the two groups in the demographic data of operation, including age, sex, Garden type of fracture (garden III/IV type), and the time from injury to operation (p > 0.05). The last follow-up time was 12 months. The dates are listed in Table 1.

**Table 1:** Characteristics and hospitalization information of patients

Variables	FNS	CCS	Statistical values	*p*
Case	42	45		
Age (years)	47.3 ± 6.8	49.1 ± 7.5	1.170 ▴	0.245
Gender (male/female)	18/24	20/25	0.022 △	0.887
Left/right	20/22	22/23	0.014 △	0.906
Time from injury to surgery (hour)	34.8 ± 5.8	36.4 ± 6.4	1.219 ▴	0.226
Garden III/IV	31/11	30/15	0.529 △	0.467
Pauwel’s angle (°)	58.6 ± 4.2	59.4 ± 5.5	0.758 ▴	0.450
Hospital stay (days)	7.5 ± 1.3	8.6 ± 1.6	3.504 ▴	< 0.001 **
Hospitalization cost (dollars)	6322.4 ± 562.6	5672.5 ± 438.5	6.032 ▴	< 0.001 **

The data are shown as *n* or mean ± standard deviation. ▴ t values. △ Chi-squared values. * < 0.05. ** < 0.001

### Surgical outcomes

**Fig. 3:**
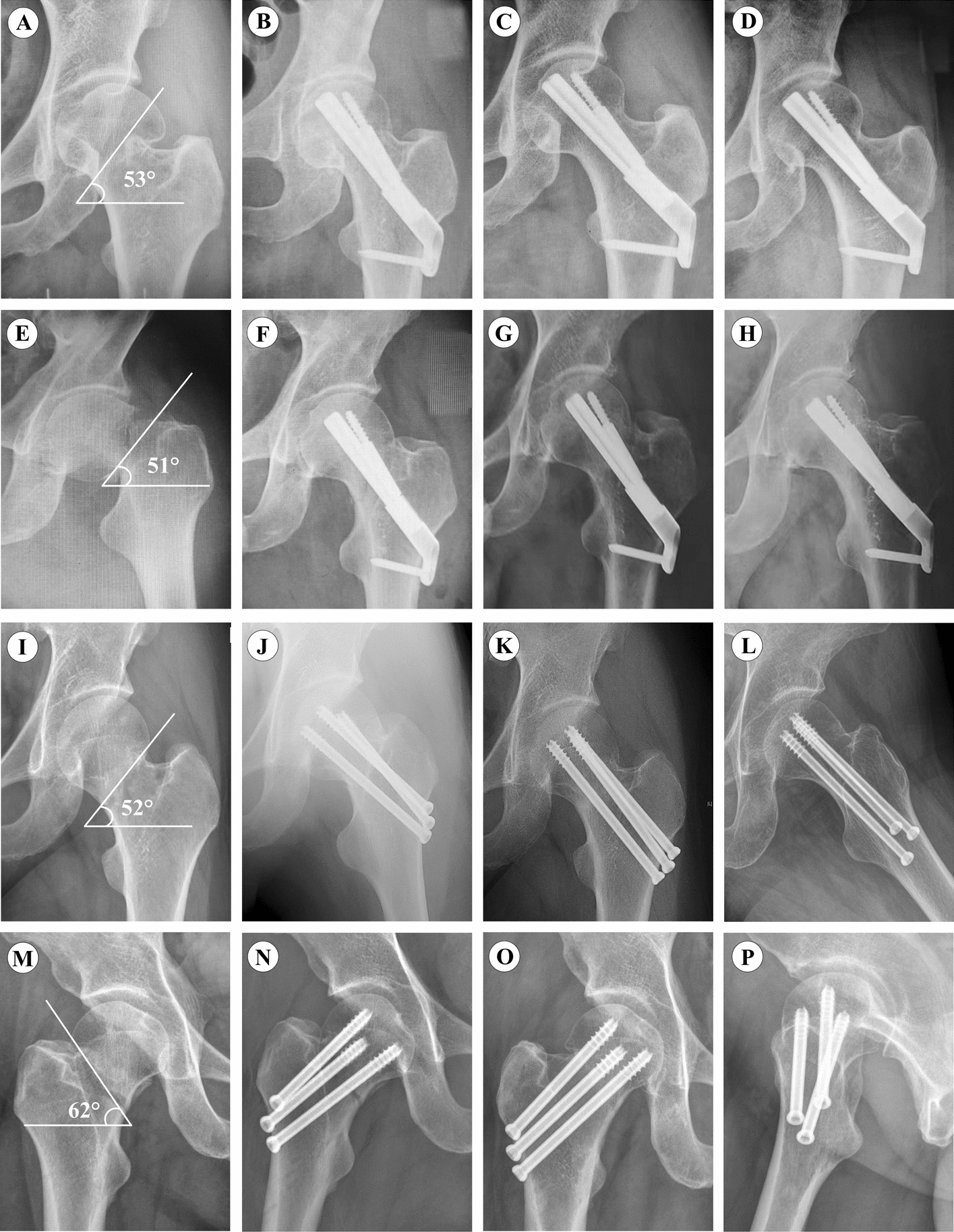
Radiographs for FNS and CCS groups. **A** Preoperative anteroposterior radiograph of a 45-year-old male Pauwels III femoral neck fracture. **B** The patient received anteroposterior X-ray with FNS one day after surgery. **C** Anteroposterior radiograph at 3-month follow-up after FNS. **D** Anteroposterior radiograph at 12-month follow-up after FNS; **E** Preoperative anteroposterior radiograph of Pauwels III femoral neck fracture in a 48-year-old woman. **F** The patient received anteroposterior X-ray with FNS one day after surgery. **G** Anteroposterior radiograph at 3-month follow-up after FNS. **H** Anteroposterior radiograph at 11-month follow-up after FNS use; **I** Preoperative anteroposterior radiograph of Pauwels III femoral neck fracture in a 53-year-old woman. **J** The patient received anteroposterior X-ray with CCS 1 day after surgery. **K**, **L** Anteroposterior and axial radiograph at 6-month follow-up after CCS; **M** Preoperative anteroposterior radiograph of a 52-year-old male Pauwels III femoral neck fracture. **N** Anteroposterior radiograph with CCS one day after surgery. **O** The patient received anteroposterior X-ray with FNS one day after surgery. **P** Lateral radiograph at 6-month follow-up after CCS

There was no significant difference in the time from fracture to operation and the amount of blood loss (hemoglobin loss) between the two groups (*p* > 0.05). The operation time and fluoroscopy time of FNS were significantly shorter than those of CCS (*p* < 0.001). Harris scores were obtained by assessing the degree of pain, daily activity, and range of exercise. After 6 and 12 months of follow-up, the Harris score of FNS was significantly better than that of CCS in the VAS score. The postoperative healing time of FNS = 12.4 ± 1.7 weeks was shorter than that of CCS = 14.3 ± 1.4 months (*p* < 0.001). Typical cases are shown in Fig. 3.

#### Complications

After 12 months of follow-up, 40 patients in the FNS group and 39 patients in the CCS group (*p* = 0.312) had a bony union. There was a case of femoral head necrosis in the FNS group and two cases of femoral head necrosis in the CCS group (*p* = 0.951). Femoral neck shortening was documented in patients with healed fractures and showed horizontal shortening (> 5 mm) in 17% of patients in the FNS group and 31% in the CCS group, *p* = 0.116. The fixation failure rates (screw loosening and screw pull-out) were 0% and 18%, respectively, *p* = 0.012.

Surgery and postoperative follow-up information of patients are listed in Table 2.

**Table 2:** Surgery and postoperative follow-up information of patients

Variables	FNS	CCS	Statistical values	p
Operation duration (min)	62.2 ± 8.2	77.4 ± 9.2	8.113 ▴	< 0.001 **
Hemoglobin loss (g/L)	14.6 ± 6.3	16.5 ± 6.7	1.360 ▴	0.177
Duration of fluoroscopy (seconds)	42.5 ± 8.6	56.7 ± 7.1	8.421 ▴	< 0.001 **
Harris score (3 month)	66.5 ± 3.4	67.4 ± 4.1	1.110 ▴	0.270
6 month	76.3 ± 5.4	73.4 ± 5.2	2.551 ▴	0.012 *
12 month	81.1 ± 4.6	77.4 ± 3.6	4.193 ▴	< 0.001**
Healing time (weeks)	12.4 ± 1.7	14.3 ± 1.4	5.706 ▴	< 0.001**
Fixing failure (screw loosening/ screw out)	0(0%)	8(18%)	6.231 △	0.012*
Femoral neck shortening	5(12%)	14(31%)	4.695 △	0.030*
< 5 mm	35(83%)	31(69%)	2.475 △	0.116
5–10 mm	6 (14%)	11 (24%)	1.426 △	0.232
> 10 mm	1(3%)	3(7%)	0.1950△	0.659
Nonunion	1	4	0.770 △	0.399
Femoral neck necrosis	1	2	0.004 △	0.951

The data are shown as *n* or mean ± standard deviation. ▴ t values. △ Chi-squared values. * < 0.05. ** < 0.001

## Discussion

Vertical femoral neck fractures are mostly caused by high-energy trauma [17, 18]. Such fractures are susceptible to high shear stresses due to the anatomical characteristics of the femoral head and neck. This complicates their management and increases the risk of complications, such as osteonecrosis of the femoral head and neck, nonunion, and these problems [19]. Compared to small angle fractures, it requires stronger fixation to achieve stable fracture healing. Therefore, strategies to optimize fixation stability in patients with vertical femoral neck fractures remain controversial [20].

From finite element analysis, we found that the FNS device showed lower displacement than CCS when Pauwels angles were 55° and 65°, indicating that FNS has higher stability than CCS in common vertical fractures. The internal fixation stress of FNS was higher than that of cannulated screws regardless of the fracture line of vertical fractures. Regarding VMS, about 1.5–3.0 times that of the cannulated screw group, which was the same as Fan's previous study [21]. The VMS in FNS was concentrated at the junction of the sliding hip screw and the anti-rotation screw, which means that FNS may have a higher risk of internal implant fracture than cannulated screws. However, no cases of internal fixation fractures have been identified clinically. In addition, because FNS has the ability to bypass femoral shaft loads and more implant load distributions, CCS has the most stress dissipation at the fracture site. Repeated cyclic loading at the fracture site makes it easier for CCS to reach fatigue failure of the implant, a result that predicts fixation failure. In summary, FNS has the potential to perform better than CCS in patients with common vertical fractures (Pauwels angle < 65°).

Previously, FNS was compared with a variety of internal fixation for clinical outcomes. Tang mentioned in the comparison of FNS with inverted hollow cancellous screws that angular fixation devices may have better resistance to varus deformity and micromotion than traditional inverted triangular screws. FNS has shown promising clinical results in resisting femoral neck shortening and complications [22]. Hu also concluded in a clinical comparison between FNS and hollow compression screws that FNS has excellent biomechanical properties and significantly improved overall structural stability [23]. Stoffel also demonstrated from biomechanical experiments that FNS was more stable than the cannulated cancellous screw group under load testing. Schopper et al. concluded that FNS was more resistant to varus deformity than the Hansson screw system for Pauwels III fractures [15].

Femoral neck shortening after internal fixation of femoral neck fractures is a well-known phenomenon, especially in high shear forces such as the vertical femoral neck. In our study, 83% of patients in the FNS group had no/mild shortening (< 5 mm), 14% had moderate shortening (5–10 mm), and 3% had severe shortening (> 10 mm), while 69% of patients in the CCS group had no/mild shortening (< 5 mm), 24% had moderate shortening (5–10 mm), and 7% had severe shortening > 10 mm, and FNS had better resistance to femoral neck shortening than CCS. Previous studies have also reported a high incidence of femoral neck shortening after cannulated screw fixation. Zlowodski et al. reported a shortening rate of 31% for undisplaced fractures and 27% for displaced fractures [24]. Slobogean et al. reported moderate and severe shortening in more than 30% of patients under 55 years of age receiving multiple cannulated screw instrumentation [25]. FNS anti-rotation screws are cross-fixed between dynamic rods, providing angular stability and preventing screw retropulsion [14, 15]. Jun-Ki Moon et al. found that FNS fixation can provide biomechanical stability comparable to DHS [26]. We found that screw withdrawal was not observed in the FNS group, whereas fixation failure (screw loosening and screw pull-out) was observed in the CCS group, and the difference was statistically significant (*p* < 0.05). About postoperative rehabilitation, our requirements for the patients were nonweight-bearing of the affected limb within three months after surgery and partial weight-bearing after fracture healing (walking with assistive device assistance). Appropriate weight-bearing was gradually increased 6 months after surgery. So we have not seen any internal fixation device breakage in our patient, although there may have been such a risk. Failure of internal fixation of implants is not only related to stiffness of the device but also to weight-bearing time management and the characteristics of the instrumentation that conduct the stresses imposed (i.e., fatigue failure).

This study has the following limitations. First, this study used a finite element partial simulation model that simplifies cartilage, muscle attachment, and ligaments. This was a retrospective study with a limited number of cases, so there may have been selection bias. Additionally, the preoperative reduction will not ensure that all fractures achieve the ideal state. Displacement differences may lead to a bias in complication rates. More realistic biomechanical experiments are expected to validate our results. Our findings need to be validated by a large sample size and a more extended follow-up period in future studies.

## Conclusion

In summary, this device has good anti-rotation properties to avoid femoral neck shortening and internal fixation failure after fracture healing. The FNS instrumentation system allows vertical femoral neck fractures to be treated more effectively.

## Materials and methods

### Finite element analysis

**Fig. 4:**
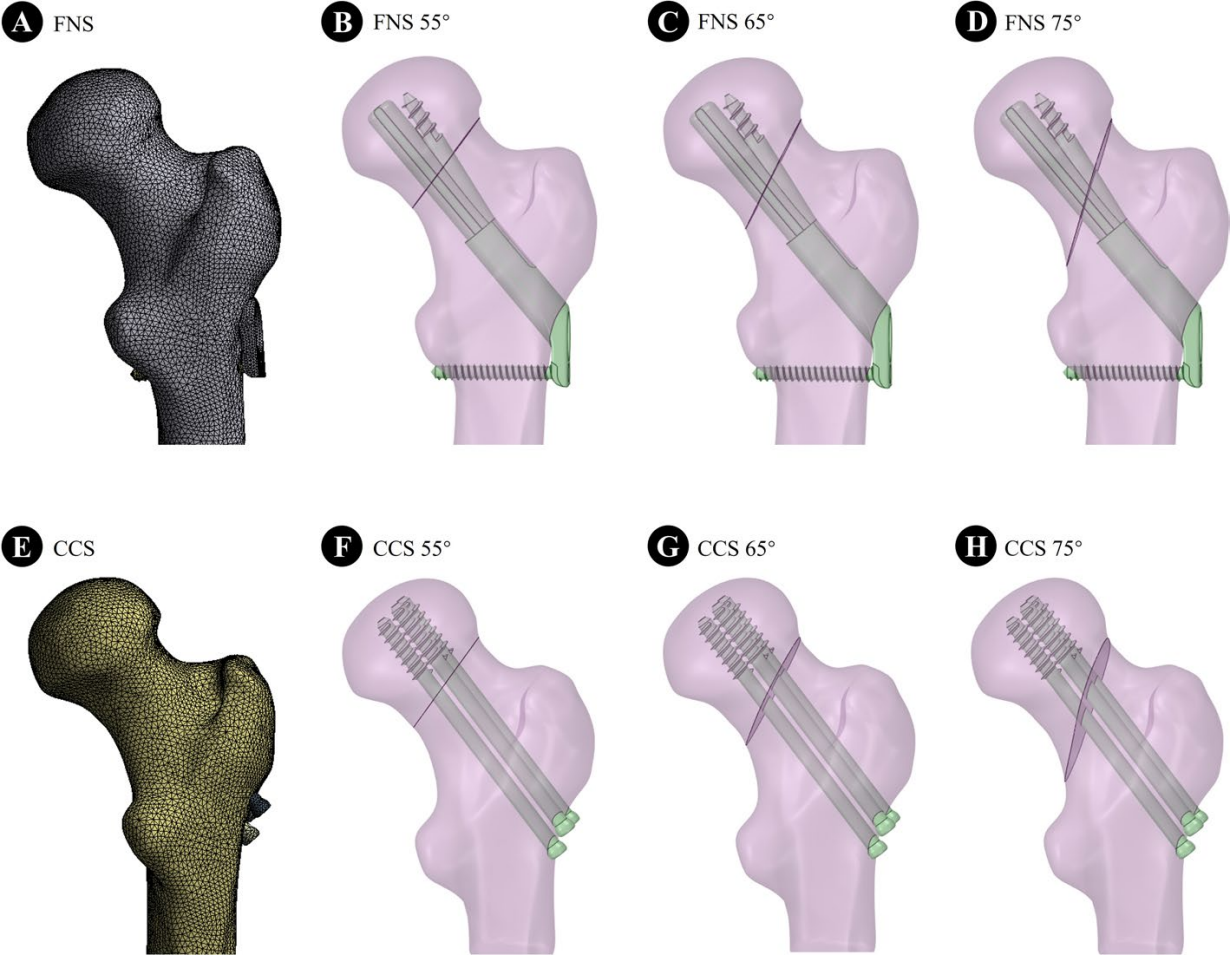
Models of femoral neck fractures with virtual fixation using FNS and CCS with Pauwels angles of 55°, 65°, and 75°, respectively

A 26-year-old male volunteer was recruited, and the femur was scanned using a Siemens 64-row CT scanner with a thickness of 0.6 mm, and a resolution of 512 × 512 pixels. The femoral CT data were extracted and imported into MIMICS 21.0 (MATERIALISE, Leuven, Belgium). A three-dimensional model of the upper femur was established based on the gray value of the tissue using the region segmentation command and then exported in stereolithography (STL) format. These STL formats were imported into the Geomagic Wrap 2017 software (Geomagic Wrap 2017, USA) for smoothing, meshing, noise reduction, and surface adaptation and then into the SolidWorks 2017 software (Dassault, France). The three-dimensional models of cortical and cancellous bone were established by Boolean operation, and the femoral neck fracture models with Pauwels angles of 55°, 65°, and 75° were established. According to the fixed clinical procedure and engineering geometry data, the models of CCS and FNS were generated by Solidworks software (Fig. 4). In the ANSYS Workbench software (ANSYS, American), each component was meshed by solid tetrahedral elements, and the grid convergence calculation was tested by different sizes. Regarding material parameters, cortical bone, cancellous bone, femoral neck plate, and locking screw were assumed to be continuous, isotropic, and uniform linear elastic materials. The parameters of each component used in the calculation and the number of nodes and units of the two assembly units are shown in Tables 3 and 4 [27, 28]. According to the contact method described in the reference, the fracture surface was set to friction (coefficient of friction = 0.46). The titanium plate and bone surface were contacted by friction (friction coefficient = 0.3); the Friction coefficient between the screw and sleeve = 0.23. The threaded screw area was used for bonding with the bone, and the nonthreaded area was kept in contact with the bone. The screws were used to contact the titanium plate [27, 29]. For calculation purposes, the distal femur was fixed entirely. To simulate the single-leg standing posture, each calculated assembly model extended 10° outward, tilted 9° backward, and was statically loaded to the center of the femoral head with a downward vertical force of 2100N [30].

**Table 3:** Material properties information consisting of finite elements models

	Titanium alloy	Cortical bone	Cancellous bone
E (GPa)	105	16.8	0.84
Poisson’s ratio	0.35	0.3	0.2

**Table 4:** Element information consisting of finite elements models

Model	Pauwels angle of 55°	Pauwels angle of 65°	Pauwels angle of 75°
FNS			
Node	363,521	428,723	498,219
Unit	278,243	288,519	314,218
CCS			
Node	270,069	289,414	301,942
Unit	184,177	192,584	220,142

### Clinical research

We retrospectively analyzed the data of 87 patients (< 65 years old) with femoral neck fractures treated in our hospital from May 2019 to May 2021. The fracture type was Pauwels III vertical femoral neck fracture (Garden Types III or IV). A total of 42 patients received FNS treatment, and 45 received CCS treatment. Inclusion criteria were (I) patients with vertical femoral neck fracture less than 65 years old; (II) patients treated with FNS or CCS; and (III) patients who completed follow-up for at least one year. The exclusion criteria are as follows: (I) femoral head fracture with other ipsilateral or contralateral lower limb fractures; (II) patients with an insufficient reduction during operation; and (III) other fractures or other diseases (pathological fractures and rheumatoid diseases) that affect the treatment of the femoral neck. All patients followed similar postoperative treatment regimens: anticoagulants were administered 24 h after surgery to prevent lower extremity deep venous thrombosis. The affected limb did not bear weight within three months after the operation, partially loaded after fracture healing (walking with the aid of an assistive device). All were loaded six months after the operation.

All patients were treated with FNS or CCS voluntarily. This study was approved by the institutional review board, and all patients signed an informed consent form.

## Postoperative management

The procedure of the operation refers to our previous research [16]^.^ The operation time, hemoglobin loss, and fluoroscopy time of the two groups were recorded. After the operation, patients were given oxygen, ECG monitoring, nutritional support, and sufficient antibiotics to prevent infection until the condition was stable. Each patient was followed up clinically and radiologically at 3, 6, and 12 months after the operation to evaluate the quality of life, hip function, and complications scientifically. Complications included fracture nonunion, osteonecrosis of the femoral head, shortening of the femoral neck, and internal fixation failure. Hemoglobin loss is expressed as preoperative hemoglobin minus hemoglobin on the second day after the operation. Nonunion is defined as the persistence of the fracture line six months after the operation. Femoral neck shortening was measured as described by Zielinski et al. and evaluated in the horizontal plane (abductor moment arm shortening) and vertical plane (femoral length reduction). The internal fixation failure types include screw prolapse, screw loosening, and screw pull-out [19, 31].

## Data Availability

The datasets used and/or analyzed during the present study are available from the corresponding author upon reasonable request.
